# Soil microbial diversity and community composition during conversion from conventional to organic agriculture

**DOI:** 10.1111/mec.16571

**Published:** 2022-07-11

**Authors:** Sophie Q. van Rijssel, G. F. (Ciska) Veen, Guusje J. Koorneef, J. M. T. (Tanja) Bakx‐Schotman, Freddy C. ten Hooven, Stefan Geisen, Wim H. van der Putten

**Affiliations:** ^1^ Department of Terrestrial Ecology Netherlands Institute for Ecology (NIOO‐KNAW) Wageningen The Netherlands; ^2^ Department of Soil Chemistry and Chemical Soil Quality Wageningen University & Research Wageningen The Netherlands; ^3^ Laboratory of Nematology Wageningen University Wageningen The Netherlands

**Keywords:** agriculture, bacteria, fungi

## Abstract

It is generally assumed that the dependence of conventional agriculture on artificial fertilizers and pesticides strongly impacts the environment, while organic agriculture relying more on microbial functioning may mitigate these impacts. However, it is not well known how microbial diversity and community composition change in conventionally managed farmers' fields that are converted to organic management. Here, we sequenced bacterial and fungal communities of 34 organic fields on sand and marine clay soils in a time series (chronosequence) covering 25 years of conversion. Nearby conventional fields were used as references. We found that community composition of bacteria and fungi differed between organic and conventionally managed fields. In the organic fields, fungal diversity increased with time since conversion. However, this effect disappeared when the conventional paired fields were included. There was a relationship between pH and soil organic matter content and the diversity and community composition of bacteria and fungi. In marine clay soils, when time since organic management increased, fungal communities in organic fields became more dissimilar to those in conventional fields. We conclude that conversion to organic management in these Dutch farmers' fields did not increase microbial community diversity. Instead, we observed that in organic fields in marine clay when time since conversion increased soil fungal community composition became progressively dissimilar from that in conventional fields. Our results also showed that the paired sampling approach of organic and conventional fields was essential in order to control for environmental variation that was otherwise unaccounted for.

## 1 INTRODUCTION

Technological innovations in the application of mineral fertilizers, chemical pesticides, novel crop varieties and soil tillage have enhanced the productivity on arable land (Dunwell, 2013; Vitousek et al., 2009). Increased productivity by intensified agricultural practices, however, has decreased the contributions of soils and soil biota to a variety of ecosystem services, such as decomposition and suppression of soil‐borne diseases (Allan et al., 2015; Duru et al., 2015; Foley et al., 2005). The result is that conventional agriculture is increasingly dependent on external chemical inputs for fertilization, crop protection, and irrigation, leading to pollution of surface waters, greenhouse gas emission and further loss of aboveground and belowground biodiversity (Bommarco et al., 2013; Matson et al., 1997; Tsiafouli et al., 2015; Vitousek et al., 2009).

Improving the sustainability of agricultural ecosystems might involve switching to organic management because then nutrients are released more gradually from decomposing organic fertilizers, preventing losses via leaching or gas production (Pandey et al., 2018). In addition, organic fertilizers may promote soil conditions by enhanced formation of soil organic matter and soil structure. However, organic fertilizers need to be mineralized before nutrients become available for uptake by plants. Soil microorganisms play an important role in mineralization of organic fertilizers and crop residues and it may be expected that switching from conventional to organic management is followed by a transition in the soil microbial community. While such transitions have been reported from long‐term field experiments (Francioli et al., 2016; Hartmann et al., 2015; Lupatini et al., 2017), little is known about whether this also applies to farmers' fields.

Compared to intensive conventional agricultural management, which has a major impact on soil community composition and diversity (Maeder et al., 2002; Tsiafouli et al., 2015), organic management can enhance abundance, diversity and activity of bacteria and fungi, which could be driven by a higher diversity and quantity of organic inputs (Francioli et al., 2016; Hartmann et al., 2015; Lori et al., 2017; Lupatini et al., 2017; Sun et al., 2016; Verbruggen et al., 2010). Organic management or application of manure generally leads to an increase of saprotrophic and mycorrhizal fungi, whereas mineral fertilizers generally reduce the fungal to bacterial ratio (Bebber & Richards, 2022; Francioli et al., 2016; Hartman et al., 2018, 2015). While this phenomenon is mostly known from few single long‐term experimental farms with highly similar physicochemical properties, it has been poorly studied in real farmers' fields, where many other additional factors, such as soil abiotics and specific management practices, may vary as well. Different confounding factors could reduce the detectability of effects of organic management on soil microbial community composition.

Comparisons of long‐term organic and conventional fields have revealed differences in bacterial and fungal diversity and composition (Hartman et al., 2018, 2015; Lentendu et al., 2014; Lupatini et al., 2017; Potter et al., 2022). However, little is known about the rate at which these changes occur. Some studies suggest that differences start to occur in 2–4 years following transition from conventional to organic management (Briar et al., 2007; Verbruggen et al., 2010). Another study suggests that it may take at least 10 years before soil microbial composition changes following land use conversion (Schrama et al., 2018). Following up from long‐term experiments and studies of other systems like nature restoration areas (Morriën et al., 2017), the challenge is to determine whether and at what rate such belowground transitions are occurring in farmlands. Temporal developments in soil might be tested by a space‐for‐time substitution approach using a time series (chronosequence) of a number of different fields with different times since conversion. Such approaches have been taken in order to examine shifts in microbial composition during saltmarsh succession (Schrama et al., 2012) and during secondary succession on abandoned agricultural land (Morriën et al., 2017). However, studies of organic agriculture chronosequence approaches including conventional references for each field, and controlling for soil type, are rare, if not absent.

Agricultural management may affect abiotic parameters such as pH and soil organic matter content (Marriott & Wander, 2006; Parham et al., 2002). As microbial communities are known to be fundamentally structured by particularly pH and soil organic matter content, management differences might result in differences in microbial diversity and community composition (Lauber et al., 2008; Mulder et al., 2005; Rousk et al., 2010; Thomson et al., 2015). Many of those studies have been carried out along large‐scale environmental gradients, such as those across continents. At smaller spatial scales, for example within agricultural fields, pH has also been identified as an important factor explaining bacterial, and also to some extent fungal diversity and composition (Bainard et al., 2016; Orr et al., 2015; Wakelin et al., 2008; Zhalnina et al., 2015). Therefore, both pH and soil organic matter content might explain soil microbial composition and diversity patterns, thereby being involved as (co‐) determinants of management‐induced changes in soil community composition.

The aim of the present study was to test how diversity and community composition of bacteria and fungi shift during the conversion from conventional to organic agriculture and whether this is due to possible associated changes in pH and soil organic matter content after conversion to organic farming. We tested the hypotheses that (1) richness and Shannon diversity (alpha diversity) of bacteria and fungi is higher in organically managed agricultural fields than in conventionally managed fields, and that (2) community composition (beta diversity) of bacteria and fungi differs between organic and nearby conventional agricultural fields. Furthermore, we tested the hypothesis (3) that alpha and beta diversity increased with time since conversion from conventional to organic agricultural management. Finally, we analysed how soil organic matter content and pH affected alpha and beta diversity of microorganisms in the conventional and organically managed soils.

We tested our hypotheses for two different soil types, that is, marine clay and sandy soils. These are two major soil types in the Netherlands where agriculture is performed and we determined whether our hypotheses might depend on the type of soil examined. In order to test our hypotheses, we collected soil samples from farms that differed in time since conversion from conventional to organic farming and we performed Illumina MiSeq amplicon sequencing targeting bacteria and fungi. In order to have a proper control, a conventional field was examined nearby each organic field. In all soil samples, pH and soil organic matter content were measured in order to determine how these would predict patterns in soil microbial community composition.

## 2 MATERIALS AND METHODS

### 2.1 Site selection

We established two time series (chronosequences), one on sand and one on marine clay soils, of arable fields that have been converted from intensive conventional arable farming to organic arable farming. The chronosequences ranged from fields that had been converted 0–25 years ago. Each organic field was paired with a nearby conventional field that fit the selection criteria in order to obtain a control that was subjected to the same local variation in environmental factors. This resulted in a total of 11 pairs of organic and conventional fields on sandy soil and 23 pairs of organic and conventional fields on marine clay soil, so in total 68 fields were sampled. Organic fields all had a SKAL certificate (“Stichting Keur Alternatief voortgebrachte Landbouwproducten” which is translated as “Control authority for organic production”), based on European legislation. This includes that minimally 70% of the fertilizer is certified organic animal manure, plant materials or compost. Furthermore, there are restrictions in the use of mineral fertilizers and pesticides.

We had four selection criteria: (1) Standardized soil type: sandy soils were Anthrosols with a very low elutriable fraction and an A‐horizon of at least 30 cm, whereas marine clay soils were Fluvisols from marine origin with an elutriable fraction of 17.5%–45%. (2) Standardized crop: the fields must be covered by a monocotylous crop, preferably winter wheat (*Triticum aestivum*), and if not possible, a grass‐clover mixture or luzern. (3) Crop rotation: each field was part of a wider crop rotation with tuber crops (potatoes/onions). (4) Soil tillage: inversion tillage must have been applied at least once in the last 5 years. Locations are added in the supplement in a way that respects the privacy of the farmers involved in this research (Figure S1). Within pairs, samples were taken minimally 40 m apart (in the case of neighbouring fields) and maximally 13 km. The maximal distance between the most extremely situated fields within soil type was about 140 km.

### 2.2 Soil sampling and analysis of chemical properties

Within each field we collected soil samples from three subsites that were situated minimally 30 m from the field edge and approx. 2 m from tractor tracks. Subsites were minimally 15 m apart. At each sampling subsite, we collected a soil sample of approximately 3 kg from 5–15 cm depth. The topsoil was removed because processes in the topsoil may be largely driven by variations in daily weather conditions. Soil samples were stored at 4°C. Within a week soil samples were gently homogenized and a subsample of 6 g was sieved using a 4 mm mesh to remove coarse elements. From this subsample, we stored 1 g of soil in an Eppendorf tube at −80°C for analysis of the soil microbial community composition. The remaining soil was stored at field moisture at 4°C. From this soil we used a subsample to determine soil organic matter (SOM) content by loss‐on‐ignition. Samples were dried at 105°C and then placed in a muffle furnace for 8 h at 430°C. soil organic matter content was calculated as the weight difference between samples heated at 105 and 430°C, respectively. Another subsample was used to determine pH by shaking 10 g dry weight equivalent fresh soil in 25 ml of demi water for 2 h at 250 rounds/min (Mettler Toledo). Chemical analyses were done within 2 months after collection.

### 2.3 DNA extraction and amplification

To identify the alpha and beta diversity of bacteria and fungi, DNA was extracted from 0.25 g bulk soil using the DNeasy PowerSoil Kit (Masella et al., 2012) (Qiagen) according to the manufacturer's instructions. Community composition of bacteria was determined by amplifying the V4‐region of the 16S rRNA gene using 515f and 806 rbc primers (Caporaso et al., 2012) and the fungal community composition was determined by targeting the ITS2 region using ITS4 (reverse) and fITS9 (forward) primers (Ihrmark et al., 2012; White et al., 1990). Polymerase chain reactions (PCRs) were performed in reaction mixtures containing 15.6 μl MQ, 0.15 μl fast start high fidelity PCR system (Roche), 2.5 μl FastStart high fidelity reaction buffer with 18 mM MgCl_2_ (10× concentrated), 2.5 μl 2 mM dNTP's, 1 μl 25 mM MgCl_2_ Stock Solution, 1.25 μl BSA (4 mg/ml) and 0.5 μl of forward and 0.5 μl of tagged reverse primer and 1 μl genomic DNA. The PCR conditions were as follows: an initial denaturation step of 94°C for 5 min, 35 cycles of 45 s at 94°C, 60 s at either 51°C (16S) or 54°C (ITS) and 90 s at 72°C, followed by a final extension step for 10 min at 72°C. We purified the PCR products using Agencourt AMPurebeads (Beckman Coulter) using a ratio of 1:0.7 of PCR product to bead volume. The purification was carried out according to the manufacturer's protocol and purified products were diluted in 30 μl MQ‐water. We then measured the concentrations of the purified PCR products with a fragment analyser (Advanced Analytical) using the standard sensitivity NGS fragment analysis kit (Advanced Analytical). The products were mixed in equal nano g quantities and sent to BGI for 250 bp paired‐end sequencing with Illumina MiSeq.

### 2.4 Sequence processing

After sequencing, the raw reads were filtered. Data filtering includes removing adaptor sequences, contamination and low‐quality reads from raw reads. This was done by the sequencing company BGI. The 16S rRNA gene amplicon reads were analysed using the dada2 pipeline (version 1.18) (Callahan et al., 2016) using truncLen 250 for forward reads and 200 for reversed reads (maxN = 0, maxEE = c[2,2], truncQ = 2, rm.phix = TRUE). Consensus method was used to remove chimeric sequences. Taxonomy was assigned using the SILVA SSU database (version 138) (Quast et al., 2012).

ITS sequences were analysed using the PIPITS pipeline (version 2.4, standard settings) (Gweon et al., 2015) with VSEARCH implemented to pair sequences. The ITS2 region was extracted using ITSx (Bengtsson‐Palme et al., 2013) and short reads (<100 bp) were removed. Chimeric sequences were removed by comparing with UNITE UCHIME database (version 8.2) (Edgar et al., 2011). Sequences were aligned and classified using with RDP against the UNITE fungal database (Kõljalg et al., 2013; Nilsson et al., 2019).

### 2.5 Sequence data preparation

Low abundant amplicon sequence variants (ASVs) with 3 or fewer reads were removed as they are potentially sequencing artefacts (Auer et al., 2017). As well, we removed ASVs that occurred in ≤ than three samples. In 191 out of 204 16S and in 183 out of 195 ITS samples were included in downstream analyses.

### 2.6 Data analyses

We analysed the original number of reads, diversity and composition of bacterial and fungal data separately. For each sample we calculated the original number of reads before rarefying or normalizing. To test whether number of reads, ASV richness and Shannon diversity were affected by soil type (sand vs. marine clay) and management (organic vs. conventional) we first calculated the average of each field, and used that as a dependent variable in the linear models. Similar models were applied to describe pH and soil organic matter content differences between soil types and management.

Before calculating observed ASV richness and Shannon diversity index we removed non‐targeted ASVs and rarefied the data (16S to 5000 reads per sample; ITS to 1500 reads per sample). Rarefaction curves of 16S and ITS are presented in Figure S2. We checked our rarefaction using bootstrapping by running the rarefaction 1000 times before testing the effect of management and time on richness and diversity for all groups. This resulted in the same results, or at least the same trend, in 97%–100% of the rarefactions, indicating that the results we obtained were quite robust. For compositional analysis we first normalized the 16S and ITS data using cumulative sum scaling. We used principal coordinate analysis (PCoA) based on the Bray–Curtis dissimilarity matrix to perform unconstrained ordination. This was done on individual sample level, but then we averaged the PCoA coordinates at the field level. All subsequent analyses were run at field level, were we used PCoA axes as response variables.

To test differences in microbial community composition between regions (Zeeland and Flevoland for marine clay and Drenthe/Overijssel, Gelderland and Brabant/Limburg for sand; samples were located within or on the border of these Dutch provinces) using analyses of variance (ANOVA's) and a Tukey's post‐hoc test on both PCoA axes. However, since each region included both older and younger organically managed fields we did not do separate analyses per region. Instead, we performed separate analyses for marine clay and sandy soils and for the models run on marine clay soils we included region as a random factor. Within sand we did not find differences in composition between regions.

We used linear mixed models (marine clay) and linear models (sand) to test the relationship between alpha or beta diversity and time since conversion, pH and soil organic matter content using management as covariable. To control for potentially unobserved factors underlying time since transition, we assigned the same time since conversion of organic farms to their paired conventional farm. In addition, we constructed a linear mixed effects model with pH and soil organic matter content as explanatory variables of microbial richness and diversity. Here we used forward model selection based on AIC (Akaike, 1974) to determine which factors to include in the model making the models not more complex than needed.

To test if beta diversity between organic and conventional farms increased over time, we also calculated the average Bray–Curtis dissimilarity within each pair of organic and conventional fields. We then used the average dissimilarity as a response, and time as an explanatory variable in a linear model.

We tested the correlations between time since conversion and pH and soil organic matter for both organic and conventional fields. We assigned the same time since conversion of organic farms to their paired conventional farm. We assessed this by linear mixed effects models (marine clay) and linear models (sand) with time as explanatory variable with region as random factor (marine clay).

All statistical analyses were performed in R (version 4.1.1) (R Core Team, 2021). We used the phyloseq package (version 1.36.0) for filtering and rarefying our data (McMurdie & Holmes, 2013) and the metagMisc package (version 0.0.4) (Mikryukov, 2019) and metagenomeSeq package (version 1.34.0) for normalization (Paulson et al., 2013). Bootstrapping was performed using a “for loop”; running 1000 times the rarefaction and subsequent analyses; R code can be found in Supporting Information Materials. Linear (mixed) models were performed using the package nlme (Pinheiro et al., 2021). We used type I analysis of variance. PCoA axis scores were performed using the ape package (version 5.5) (Paradis et al., 2004). Figures were made with ggplot2 (version 3.3.5) (Wickham, 2016).

## 3 RESULTS

### 3.1 General patterns

#### 3.1.1 Reads and ASVs

The average number of reads per sample was 16,482 ± 632 for bacteria (max = 72,590; min = 4842) and 7196 ± 271 for fungi (max = 23,898, min = 1580). In total, our data set contained 6209 bacterial and 3331 fungal ASVs (taxa) and 36 bacterial and 13 fungal phyla. All subsequent analyses were performed at the field level, because the three samples from one field are pseudo replicates. For bacteria and fungi neither soil type nor management caused differences in the number of reads (*F*_1,63_ < 2.2, *p* = .15).

#### 3.1.2 pH and soil organic matter content

The fields on sand on average had lower pH (6.04 ± 0.11) than on marine clay (7.99 ± 0.02; *F*_1,63_ = 563*, p* < .001), but variance was higher (Levene's test: *F*_1,65_ = 50.85*, p* < .0001). Soil organic matter content of fields on sand was higher (4.3 ± 0.13%) than on marine clay (3.4 ± 0.13%; *F*_1,63_ = 11.9*, p* < .001). Variance in soil organic matter content was also higher on sand than on clay (Levene's test: *F*_1,65_ = 5.47, *p* = .02245). Both pH and soil organic matter content were not explained by conventional versus organic management (pH: *F*_1,63_ = 1.13*, p* = .292; SOM: *F*_1,63_ = 0.26*, p* = .606), independent of soil type (pH: *F*_1,63_ = 0.01*, p* = .932; SOM: *F*_1,63_ = 0.60*, p* = .440). Variances of pH and soil organic matter content were not significantly different between conventional and organic management (Levene's test; pH: *F*_1,65_ = 0*, p* = .998; SOM: *F*_1,65_ < 0.001, *p* = .98).

#### 3.1.3 Effects of soil type on alpha and beta diversity

Fungal richness and Shannon diversity were higher on sand (Figures S3 and S4; Richness: 202 ± 5 ASVs for sand and 180 ± 3 ASVs for clay; Shannon: 4.1 ± 0.055 for sand and 3.8 ± 0.041 for clay; ANOVA: 15.32 < *F*_1,66_ < 19.23*, p* < .0003). Bacterial richness and diversity were not significantly different between fields on marine clay and sand (Figures S3 and S4; ANOVA: *F*_1,66_ < 0.64*, p* > .4).

**FIGURE 1:**
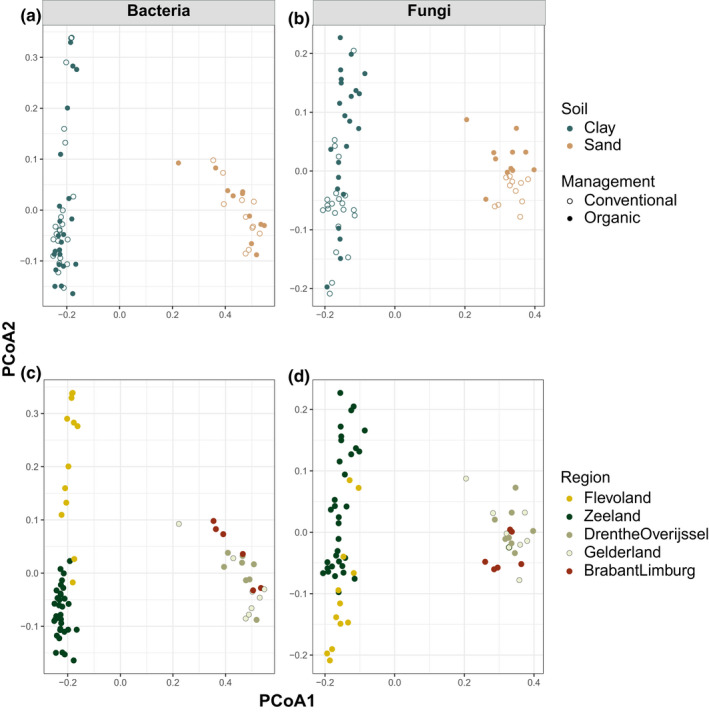
Average PCoA axis scores of bacterial (a and c) and fungal (b and d) communities based on Bray–Curtis dissimilarity. Dot colour indicates soil type (a and b)/region (c and d) and open and closed dots conventional and organic agricultural management

Composition differed between regions, for all groups along PCoA1 and PCoA2 (Figure 1; ANOVA PCoA1: *F* > 654, *p* < .0001; PCoA2: *F* > 5.9, *p* < .0001). These differences mostly were due to marine clay versus sand, only within marine clay there was also a difference between the two regions along PCoA2 (Figure 1; Tukey test marine clay: *p* < .0002; Tukey's test sand; *p* > .37).

### 3.2 Time since conversion from conventional into organic management

**FIGURE 2:**
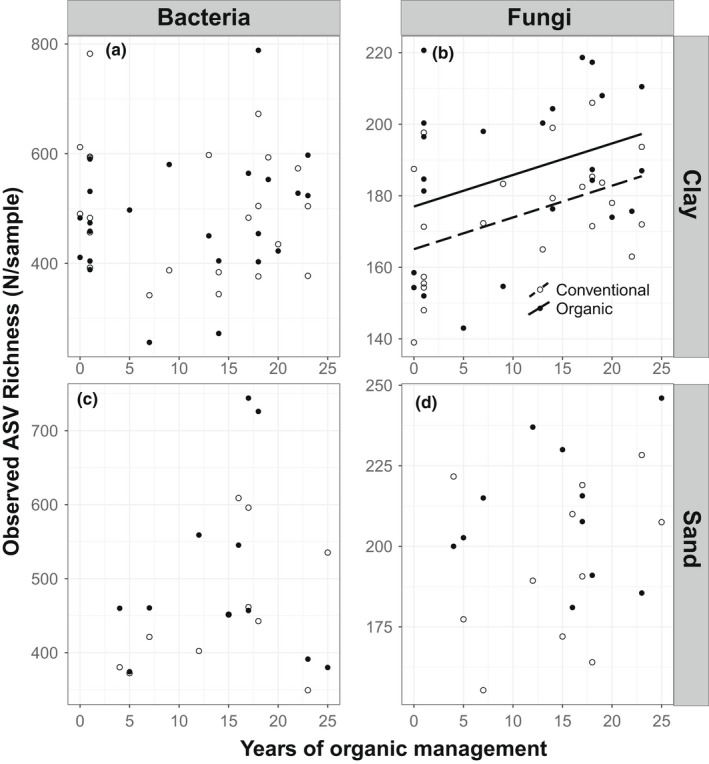
Effect of time since conversion from conventional to organic management on observed ASV richness of bacteria and fungi. Conventional fields are plotted using the time since conversion of their paired organic field. Relationships were tested using linear mixed models, with field as a random factor. Note that the scale of the y‐axis differs between bacteria and fungi because the number of ASVs in each group differs strongly. Lines are plotted for significant relationships (Table 1). Solid lines correspond to organic management, dashed lines correspond to conventional management

**FIGURE 3:**
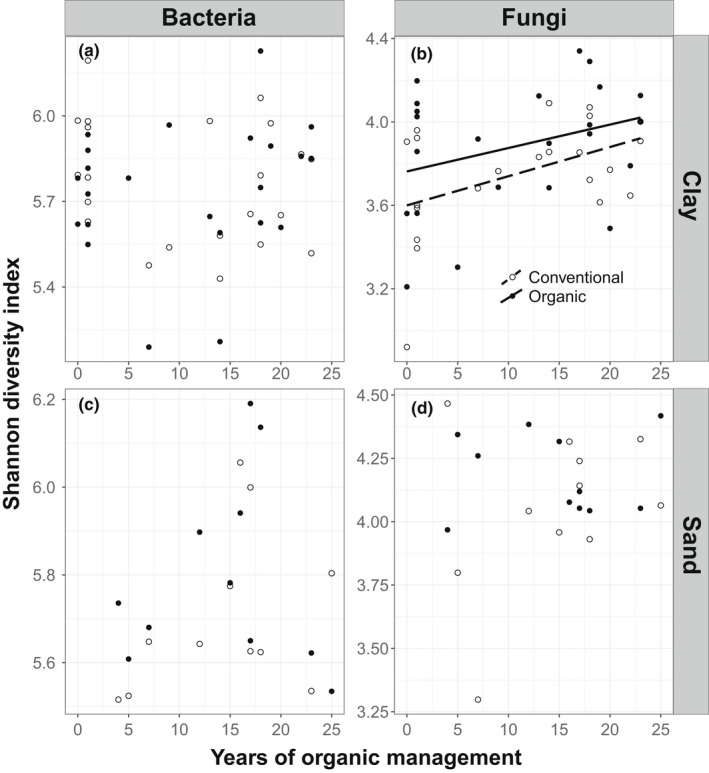
Effect of time since conversion from conventional to organic management on Shannon diversity of bacteria and fungi. Conventional fields are plotted using the time since conversion from their paired organic field. Relationships were tested using linear mixed models, with field as a random factor. Note that the scale of the y‐axis differs between bacteria and fungi because the number of ASVs in each group differs strongly. Lines are plotted for significant relationships (Table 1). Solid lines correspond to organic management, dashed lines correspond to conventional management

Bacterial ASV as well as Shannon diversity in both sand and marine clay soils were neither affected by management nor by time (Figures 2 and 3; Table 1). Fungal ASV richness and Shannon diversity increased with time since conversion on marine clay soils (Figure 2; Table 1). However, we found that this pattern did not differ among organic and conventionally managed fields, indicating that the effect of time also appeared in the conventional fields, when each of those fields was given the same time as the nearby organically managed field (Figure 3; Table 1). There was no such correlation with time since conversion and fungal community composition on sandy soils.

**TABLE 1:** The influence of soil type, management, duration of organic management and their interactions, on observed ASV richness and Shannon diversity of marine clay and sandy soils tested in linear mixed effects models with region as random factor (marine clay) and linear models (sand)

	Bacteria			Fungi		
	DenDF	*F*	*p*‐Value	DenDF	*F*	*p*‐Value
Observed ASV richness clay						
Management	40	0.31	.579	40	4.42	**.042**
Time	40	0.03	.864	40	4.38	**.043**
Management:Time	40	1.99	.166	40	0.00	.986
Observed ASV richness sand						
Management	18	1.11	.304	18	2.52	.130
Time	18	0.96	.340	18	0.97	.337
Management:Time	18	0.19	.668	18	0.28	.601
Shannon diversity clay						
Management	40	0.21	.650	40	2.71	.107
Time	40	0.00	.968	40	4.66	**.037**
Management:Time	40	1.81	.186	40	0.08	.779
Shannon diversity sand						
Management	18	1.30	.269	18	1.33	.264
Time	18	0.71	.410	18	1.33	.265
Management:Time	18	0.31	.586	18	0.87	.363

*Note*: Values in bold represent significant effects with *p* < .05. Numerator degrees of freedom is 1 for all variables.

Abbreviations: DenDF, denominator degrees of freedom (estimated with the Satterthwaite method); *F*, *F*‐value; *p*, *p*‐Value.

**FIGURE 4:**
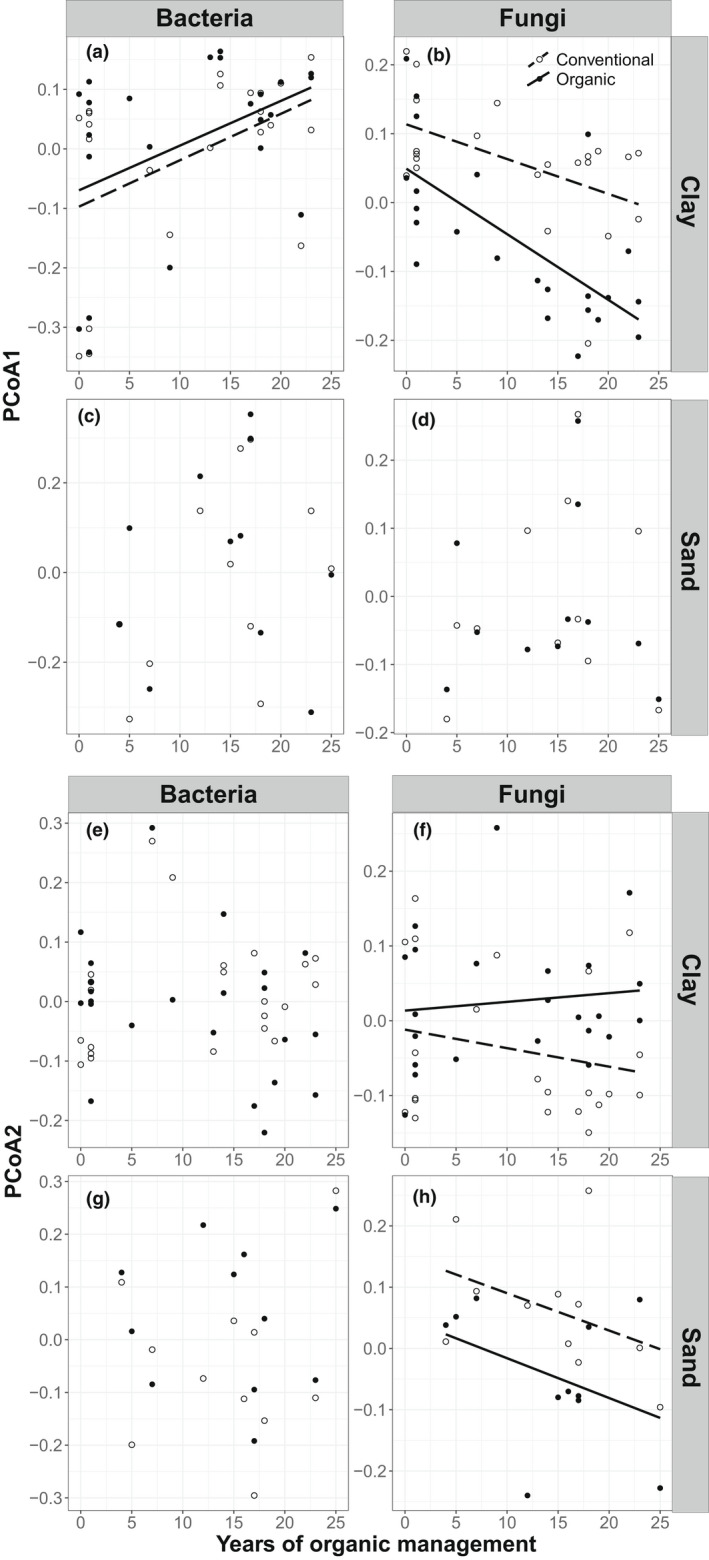
Effect of time since conversion from conventional to organic management on ASV composition of bacteria and fungi as represented by PCoA1 and PCoA2. Conventional fields were plotted using the time since conversion from their paired organic field. Relationships are tested using linear mixed models, with field as a random factor. Note that the scale of the y‐axis differs between groups because the axes differ strongly. Lines are plotted for significant relationships (Table 1). Closed dots and solid lines correspond to organic management, open dots and dashed lines correspond to conventional management

Time since conversion to organic management was correlated with bacterial and fungal community composition along the first PCoA axis (Figure 4; Table 2; *p* < .03). The first PCoA axis of fungal composition was also affected by management. For both bacterial and fungal composition, the interaction between management and time was insignificant (Figure 4; Table 2; *p* < .03). However, along PCoA2 community composition of fungi of conventional versus organically managed fields diverged with time, indicating that were longer under organic management differed more from their paired conventional fields than younger organically managed fields (Figure 4; Table 2; *p* < .05). This was supported by an additional analysis of average Bray‐Curtis dissimilarities between communities of paired fields, which was correlated with years of organic management (Figure S5; *p* = .0053).

**TABLE 2:** The influence of soil type, management, duration of organic management and their interactions, on bacterial and fungal composition of marine clay and sandy soils tested in linear mixed effects models with region as random factor (marine clay) and linear models (sand)

	Bacteria			Fungi		
	DenDF	*F*	*p*‐Value	DenDF	F	*p*‐Value
PCoA1 clay						
Management	40	0.80	.376	40	28.25	**<.0001**
Time	40	7.48	**.009**	40	21.00	**<.0001**
Management:Time	40	0.00	.983	40	3.64	.064
PCoA1 sand						
Management	18	0.22	.646	18	0.04	.842
Time	18	1.08	.312	18	0.18	.678
Management:Time	18	0.89	.357	18	0.24	.631
PCoA2 clay						
Management	40	0.58	.449	40	20.63	**.0001**
Time	40	0.11	.747	40	5.40	**.025**
Management:Time	40	3.75	.060	40	3.90	**.055**
PCoA2 sand						
Management	18	1.86	.190	18	5.88	**.026**
Time	18	0.17	.686	18	3.44	.080
Management:Time	18	0.08	.779	18	0.00	.954

*Note*: Microbial composition is summarized as PCoA axis 1 and 2. Values in bold represent significant effects with *p* < .05. Numerator degrees of freedom is 1 for all variables.

Abbreviations: DenDF, denominator degrees of freedom; *F*, *F*‐value; *p*, *p*‐Value.

### 3.3 The role of pH and soil organic matter content in time since conversion

Changes in ASV richness, diversity and community composition (alpha and beta diversity) were related to pH and soil organic matter content. However, there was no time since conversion effect on pH and SOM, so that the time‐related pattern in fungal diversity and composition could not be explained by any change in pH or soil organic matter content (Figure S6).

Bacterial ASV richness and Shannon diversity on marine clay soils were neither affected by pH, nor by soil organic matter content. On sand, however, bacterial ASV richness and Shannon diversity showed a positive relationship with increasing pH. The rate of increase was also moderately significantly affected by the 4‐way interaction of management, time, pH and soil organic matter content (Table S1). Fungal richness and Shannon diversity on marine clay decreased with pH and soil organic matter content (Table S1). Fungal ASV richness on sand was affected by an interaction between time and soil organic matter content, as well as by three‐way interaction of time, pH and soil organic matter content (Table S1).

Bacterial composition of marine clay soils along PCoA1 was related to pH and the interaction between soil organic matter and time. Along PCoA2 soil organic matter was the only significant driver (Figure 4; Figure S8; Table S2). Bacterial composition of sandy soils along PCoA1 was related to pH. Along PCoA2 on sandy soils bacterial composition was related to pH, soil organic matter content, and the two‐ and three‐way interactions between pH, and management and time (Figures S7 and S8; Table S2). Fungal composition in marine clay along PCoA2 was affected by soil organic matter content (Figures 4; Figures S7 and S8). Fungal composition in sand along PCoA1 was affected by pH and the interaction between pH and soil organic matter content (Figure S7). Fungal composition in sand along PCoA2 was related to pH and the interaction between time and soil organic matter content (Figure 4; Figures S7 and S8; Table S2).

## 4 DISCUSSION

In this study, we tested the hypothesis that there is an increase in diversity and a shift in composition of soil bacteria and fungi during the conversion from conventional to organic agriculture. We assembled two time series (chronosequences), one on sand and one on marine clay soils, of fields that have been converted from conventional to organic agricultural management for 1–25 years. We paired each organic field with the most nearby conventional field. We found a shift in fungal community composition with duration of organic management. However, for community composition of bacteria and for richness and Shannon diversity of bacteria and fungi, we did not find effects of the duration of organic management.

### 4.1 Conventional versus organic management

In contrast to our first hypothesis, we only found differences between organically and conventionally managed marine clay soils for fungal richness, but not for bacterial richness. Also, there were no differences in Shannon diversity between organic and conventionally managed soils. These results partially support previous studies, which often found higher richness and diversity of fungi and bacteria in organically managed agricultural fields or fields where farmyard manure was used as fertilizer (Francioli et al., 2016; Hartmann et al., 2015; Lupatini et al., 2017; Morugán‐Coronado et al., 2022; Schmid et al., 2018). In organically managed fields, fungal diversity is expected to be higher than in conventionally managed fields, because of the use of organic fertilizers that need to be decomposed by microbes in order to release nutrients that can be taken up by the crop plants. Such fertilizers often increase the relative abundance of fungal species that are more capable to degrade recalcitrant material such as straw, which are less suitable for decomposition by bacteria (Clocchiatti et al., 2020; Romaní et al., 2006; van der Wal et al., 2013).

In support of our hypothesis, we found that bacterial and fungal community composition differed between organic and conventional fields on marine clay soils, and fungal composition also between organic and conventional fields on sandy soils. This finding is in line with other studies (Hartman et al., 2018, 2015; Lupatini et al., 2017; Reilly et al., 2013). That we did not find an increase in fungal richness and diversity in organic fields on sands could be explained by three factors. First, there were bigger differences in specific management practices between conventional versus organic fields on clay than on sand. For instance, we observed that organically managed fields on marine clay more often were covered by grass‐clover mixtures than conventionally managed fields. This difference between conventional and organic farming practice was not observed on sandy soils. Second, there was more variation in abiotic soil properties on sandy soils, such as a larger pH gradient. Third, we had fewer replicates on sand than on marine clay. This could have resulted differences in richness, diversity and composition of the investigated kingdoms between conventional and organic management being clearer on marine clay than on sand.

### 4.2 Effect of duration of organic management

In support of our hypothesis we found that fungal community composition in clay soils shifted with duration of organic management and became increasingly different from conventional fields. Similarly, we found that bacterial and fungal diversity increased with time since the start of organic management and that bacterial community composition shifted, however, these trends were also observed in the paired conventional fields that served as controls. Shifts in fungal community composition were also observed on long‐term experimental farms, as well as in secondary succession chronosequences (Hartman et al., 2018; Hartmann et al., 2015; Lupatini et al., 2017; Morriën et al., 2017; Potter et al., 2022). Changes in fungal community composition are accompanied by changes in soil functioning, like nutrient provisioning (Köhl et al., 2014), carbon uptake (Morriën et al., 2017), decomposition rates (van der Wal et al., 2015) and overall ecosystem multifunctionality (Wagg et al., 2014). However, in contrast with earlier work we also found temporal trends for conventional fields, which shows that other factors than duration of organic management alone may have been important drivers of microbial community composition in our chronosequence.

In contrast to our hypothesis, duration of organic management was neither related to pH, nor to soil organic matter content. Therefore, the shift in fungal community composition on clay soils with duration of organic management cannot be explained by a change in soil organic matter or pH. It could be that other ‐ not tested ‐ environmental gradients are underlying the time effect we found in both organic and conventional fields. For example, not the total content of soil organic matter, but rather the quality could be affected by duration of organic management and might be important for microbial community composition. The quality of soil organic matter includes amounts of labile and stabile carbon, but also of specific components, like cellulose and lignin (Gregorich et al., 1994). A higher variety of carbon structures could lead to greater diversity of fungi because of niche partitioning (Taylor et al., 2014). Further studies are needed in order to examine the quality of soil organic matter along the duration of organic management. Alternative explanations for finding similar time effects in conventional fields that were used as a control, could be that conventional farmers close to organic farmers have adopted some of the management practices (Dessart et al., 2021; Rust et al., 2021) that are often used in organic management (de Buck et al., 2001; Morgan, 2011). This may result in conventional farms close to older organic farms applying more organic‐like practices than conventional farms close to young organic farms, resulting in similar patterns with time since conversion in both conventionally and organically managed fields.

Whereas we found that microbial community composition and diversity shifted along our chronosequence, we also recorded substantial variation among fields of the same age, which may be explained by several factors. First, the chronosequences include fields from individual farmers, who make individual management decisions on crop rotation, fertilization and tillage (de Buck et al., 2001; Tscharntke et al., 2021). Second, some farmers that consider converting towards organic farming may have already started to reduce the amount of pesticides and artificial fertilizer in preceding years, resulting in variation among recently converted farms. Finally, as most farmers convert to organic farming field by field they gain experience during the transition. This may result in recently converted fields being more successful than fields that were converted longer ago (Martini et al., 2004) and hence in less pronounced trends with time since conversion. This may have contributed to variation in the specific management practices applied and, consequently, to variation in microbial responses that could have overridden effects of duration of organic management (Brennan & Acosta‐Martinez, 2017; Orr et al., 2015). In order to fully disentangle effects of individual farmer decisions, management practices, and (soil) environmental conditions we need further studies on microbial diversity and community composition during the transition to organic farming.

Trends with time since conversion to organic management may also be partly obscured by the DNA‐based method that we have used. In spite of this method being standard in most soil microbiome studies (Francioli et al., 2016; Hartman et al., 2018, 2015), it integrates information on inactive and even dead organisms (Carini et al., 2016), so that the history of conventional agriculture may remain ‐ at least for a while – in the organic field soils. Future studies may use RNA‐based approaches to test if the patterns found are representative also in the active fraction, while considering also the biases inherent to the short life‐span and fast turnover of RNA in soil. It will also be helpful if future work shifts from using semi‐quantitative meta‐barcoding methods (Gloor et al., 2017; Morton et al., 2019) to quantitative approaches, for example by including microbial biomass obtained from PLFA analysis. This will allow to test how the actual abundance or biomass of certain microorganisms responds to changes in agricultural management (Lewe et al., 2021; Morton et al., 2019).

## 5 CONCLUSIONS

In conclusion, we found that fungal community composition in organically managed fields was becoming more divergent from the communities in conventionally managed fields with increasing duration of organic management. However, we found relatively few differences in soil bacterial and fungal richness and diversity between conventional and organic management. Further studies are needed to determine how specific management practices and other currently unknown environmental conditions may affect microbial community composition compared to effects of conventional versus organic management, the duration of organic management, pH and soil organic matter content. The surprising findings of some similar trends in converted and conventional fields shows that without a paired field design we would have drawn false‐positive conclusions. In our case the inclusion of conventional references shows that duration of organic management is probably not the driver of microbial richness, diversity and community composition in organically managed fields in The Netherlands.

## AUTHOR CONTRIBUTIONS

Guusje J. Koorneef, Wim H. van der Putten, G.F. (Ciska) Veen and Stefan Geisen developed the study concept and design; Sophie Q. van Rijssel, Guusje J. Koorneef, J.M.T. (Tanja) Bakx‐Schotman, Stefan Geisen, G.F. (Ciska) Veen and Wim H. van der Putten collected the samples and analysed soil properties; J.M.T. (Tanja) Bakx‐Schotman completed the DNA preparation; Freddy C. ten Hooven ran the pipeline; Sophie Q. van Rijssel performed the data analysis with input of G.F. (Ciska) Veen, Stefan Geisen and Wim H. van der Putten; and Sophie Q. van Rijssel, G.F. (Ciska) Veen and Wim H. van der Putten drafted the manuscript with input of all authors. All authors have read and approved the final manuscript.

## CONFLICT OF INTEREST

The authors declare no conflict of interest.

## Supporting information


Appendix S1

## Data Availability

Metadata is available in the Dryad Digital Repository: https://doi.org/10.5061/dryad.00000005t. Sequencing data have been submitted to the European Nucleotide Archive (accession number: PRJEB41305). Code availability: the source code of R for model fitting is in the supplement and will be available on GitHub at: https://github.com/SophievanRijssel/MEC‐22‐0027.R1
