# Supplementary material for: Soil microbial diversity and community composition during conversion from conventional to organic agriculture
Source: Mol Ecol. 2022 Jul 11;31(15):4017–30. doi: 10.1111/mec.16571 (PMC9545909; doi:10.1111/mec.16571)
Supplement: Supplementary file 1 — Appendix S1 [file MEC-31-4017-s001.docx]

Supplementary information belonging to:

**Soil microbial diversity and community composition during conversion from conventional to organic agriculture**

Sophie Q. van Rijssel, G.F. (Ciska) Veen, Guusje J. Koorneef, J.M. Tanja Bakx-Schotman, Freddy C. ten Hooven, Stefan Geisen, Wim H. van der Putten

**Table S1.** Influence of soil type, management, pH, SOM content and their interactions, on bacterial, archaeal and fungal observed ASV richness and Shannon alpha diversity tested in linear mixed effects models with region as random factor (marine clay) and linear models (sand). Values in boldface represent significant effects with P < 0.05. Numerator degrees of freedom was 1 for all variables; DenDF = Denominator degrees of freedom, F = F-value, P = P-value. [-] means that the factor was removed from the model after forward selection.

|  | Bacteria | | | Fungi | | |
| --- | --- | --- | --- | --- | --- | --- |
|  | DenDF | F | p | DenDF | F | p |
| *Observed ASV richness Clay* |  |  |  |  |  |  |
| Management | 36 | 0.32 | 0.576 | 37 | 6.08 | **0.019** |
| Time | 36 | 0.03 | 0.862 | 37 | 10.22 | **0.003** |
| pH.std | [-] | [-] | [-] | 37 | 7.53 | **0.009** |
| SOM.std | 36 | 1.42 | 0.240 | 37 | 10.42 | **0.003** |
| Management:Time | 36 | 1.29 | 0.265 | [-] | [-] | [-] |
| Management:pH.std | [-] | [-] | [-] | [-] | [-] | [-] |
| Management:SOM.std | 36 | 0.01 | 0.928 | 37 | 2.11 | 0.155 |
| Time:pH.std | [-] | [-] | [-] | [-] | [-] | [-] |
| Time:SOM.std | 36 | 2.06 | 0.160 | 37 | 2.59 | 0.116 |
| pH.std:SOM.std | [-] | [-] | [-] | [-] | [-] | [-] |
| Management:Time:pH.std | [-] | [-] | [-] | [-] | [-] | [-] |
| Management:Time:SOM.std | 36 | 2.88 | 0.098 | [-] | [-] | [-] |
| Management:pH.std:SOM.std | [-] | [-] | [-] | [-] | [-] | [-] |
| Time:pH.std:SOM.std | [-] | [-] | [-] | [-] | [-] | [-] |
| Management:Time:pH.std:SOM.std | [-] | [-] | [-] | [-] | [-] | [-] |
| *Observed ASV richness Sand* |  |  |  |  |  |  |
| Management | 6 | 2.35 | 0.177 | 8 | 5.43 | **0.048** |
| Time | 6 | 2.02 | 0.205 | 8 | 2.10 | 0.186 |
| pH.std | 6 | 10.85 | **0.017** | 8 | 0.98 | 0.351 |
| SOM.std | 6 | 0.04 | 0.851 | 8 | 1.95 | 0.200 |
| Management:Time | 6 | 0.03 | 0.876 | 8 | 0.18 | 0.686 |
| Management:pH.std | 6 | 0.45 | 0.530 | 8 | 4.17 | 0.075 |
| Management:SOM.std | 6 | 1.10 | 0.335 | 8 | 2.95 | 0.124 |
| Time:pH.std | 6 | 2.53 | 0.162 | 8 | 2.15 | 0.181 |
| Time:SOM.std | 6 | 0.03 | 0.863 | 8 | 9.79 | **0.014** |
| pH.std:SOM.std | 6 | 1.18 | 0.318 | 8 | 0.06 | 0.807 |
| Management:Time:pH.std | 6 | 3.83 | 0.098 | 8 | 0.71 | 0.423 |
| Management:Time:SOM.std | 6 | 1.91 | 0.217 | [-] | [-] | [-] |
| Management:pH.std:SOM.std | 6 | 0.02 | 0.894 | 8 | 1.01 | 0.344 |
| Time:pH.std:SOM.std | 6 | 0.24 | 0.644 | 8 | 7.47 | **0.026** |
| Management:Time:pH.std:SOM.std | 6 | 9.96 | **0.020** | [-] | [-] | [-] |
| *Shannon diversity Clay* |  |  |  |  |  |  |
| Management | 36 | 0.21 | 0.647 | 28 | 4.30 | **0.047** |
| Time | 36 | 0.00 | 0.968 | 28 | 11.83 | **0.002** |
| pH.std | [-] | [-] | [-] | 28 | 7.39 | **0.011** |
| SOM.std | 36 | 1.20 | 0.281 | 28 | 16.37 | **0.000** |
| Management:Time | 36 | 1.18 | 0.284 | 28 | 0.16 | 0.695 |
| Management:pH.std | [-] | [-] | [-] | 28 | 1.06 | 0.313 |
| Management:SOM.std | 36 | 0.13 | 0.723 | 28 | 1.41 | 0.245 |
| Time:pH.std | [-] | [-] | [-] | 28 | 3.78 | 0.062 |
| Time:SOM.std | 36 | 1.16 | 0.289 | 28 | 1.56 | 0.222 |
| pH.std:SOM.std | [-] | [-] | [-] | 28 | 0.08 | 0.778 |
| Management:Time:pH.std | [-] | [-] | [-] | 28 | 0.41 | 0.526 |
| Management:Time:SOM.std | [-] | [-] | [-] | 28 | 1.95 | 0.174 |
| Management:pH.std:SOM.std | [-] | [-] | [-] | 28 | 1.84 | 0.186 |
| Time:pH.std:SOM.std | [-] | [-] | [-] | 28 | 0.16 | 0.696 |
| Management:Time:pH.std:SOM.std | [-] | [-] | [-] | 28 | 3.55 | 0.070 |
| *Shannon diversity Sand* |  |  |  |  |  |  |
| Management | 6 | 2.33 | 0.178 | [-] | [-] | [-] |
| Time | 6 | 1.27 | 0.302 | 14 | 1.25 | 0.282 |
| pH.std | 6 | 10.58 | **0.017** | 14 | 0.42 | 0.530 |
| SOM.std | 6 | 0.11 | 0.747 | 14 | 0.00 | 0.974 |
| Management:Time | 6 | 0.09 | 0.772 | [-] | [-] | [-] |
| Management:pH.std | 6 | 0.01 | 0.924 | [-] | [-] | [-] |
| Management:SOM.std | 6 | 1.08 | 0.339 | [-] | [-] | [-] |
| Time:pH.std | 6 | 1.40 | 0.282 | 14 | 0.17 | 0.689 |
| Time:SOM.std | 6 | 0.59 | 0.471 | 14 | 0.00 | 0.965 |
| pH.std:SOM.std | 6 | 2.48 | 0.166 | 14 | 0.04 | 0.851 |
| Management:Time:pH.std | 6 | 2.31 | 0.179 | [-] | [-] | [-] |
| Management:Time:SOM.std | 6 | 1.01 | 0.353 | [-] | [-] | [-] |
| Management:pH.std:SOM.std | 6 | 0.00 | 0.958 | [-] | [-] | [-] |
| Time:pH.std:SOM.std | 6 | 0.19 | 0.677 | 14 | 4.49 | 0.052 |
| Management:Time:pH.std:SOM.std | 6 | 6.86 | **0.040** | [-] | [-] | [-] |

**Table S2.** Influence of soil type, management, pH, SOM content, organic matter inputs and their interactions, on bacterial, archaeal and fungal composition along PCoA1 and PCoA2 tested in linear mixed effects models with region as random factor (marine clay) and linear models (sand). Values in boldface represent significant effects with P < 0.05. Numerator degrees of freedom is 1 for all variables; DenDF = denominator degrees of freedom, F = F-value, P = P-value. [-] means that the factor was removed from the model after forward selection.

|  | Bacteria | | | Fungi | | |
| --- | --- | --- | --- | --- | --- | --- |
|  | DenDF | F | p | DenDF | F | p |
| *PCOA1 Clay* |  |  |  |  |  |  |
| Management | 33 | 1.25 | 0.272 | 28 | 24.98 | **<0.0001** |
| Time | 33 | 11.71 | **0.002** | 28 | 19.15 | **0.0002** |
| pH.std | 33 | 7.36 | **0.011** | 28 | 0.34 | 0.565 |
| SOM.std | 33 | 3.19 | 0.083 | 28 | 0.06 | 0.808 |
| Management:Time | 33 | 0.71 | 0.406 | 28 | 3.36 | 0.077 |
| Management:pH.std | 33 | 0.05 | 0.820 | 28 | 0.33 | 0.569 |
| Management:SOM.std | [-] | [-] | [-] | 28 | 0.05 | 0.724 |
| Time:pH.std | 33 | 1.00 | 0.325 | 28 | 0.13 | 0.819 |
| Time:SOM.std | 33 | 13.27 | **0.001** | 28 | 1.92 | 0.177 |
| pH.std:SOM.std | 33 | 2.09 | 0.158 | 28 | 0.63 | 0.434 |
| Management:Time:pH.std | 33 | 2.60 | 0.117 | 28 | 1.05 | 0.315 |
| Management:Time:SOM.std | [-] | [-] | [-] | 28 | 0.06 | 0.809 |
| Management:pH.std:SOM.std | [-] | [-] | [-] | 28 | 1.39 | 0.248 |
| Time:pH.std:SOM.std | [-] | [-] | [-] | 28 | 0.56 | 0.461 |
| Management:Time:pH.std:SOM.std | [-] | [-] | [-] | 28 | 1.38 | 0.249 |
| *PCOA1 Sand* |  |  |  |  |  |  |
| Management | 8 | 0.99 | 0.348 | 9 | 0.15 | 0.711 |
| Time | 8 | 4.93 | 0.057 | 9 | 0.64 | 0.444 |
| pH.std | 8 | 62.73 | **0.047** | 9 | 37.50 | **<0.0001** |
| SOM.std | 8 | 4.02 | 0.080 | 9 | 1.10 | 0.321 |
| Management:Time | 8 | 0.29 | 0.606 | 9 | 0.13 | 0.731 |
| Management:pH.std | 8 | 0.06 | 0.813 | 9 | 0.50 | 0.497 |
| Management:SOM.std | 8 | 2.82 | 0.131 | 9 | 1.38 | 0.270 |
| Time:pH.std | 8 | 0.43 | 0.531 | 9 | 1.37 | 0.271 |
| Time:SOM.std | 8 | 0.21 | 0.661 | 9 | 0.81 | 0.391 |
| pH.std:SOM.std | 8 | 2.92 | 0.126 | 9 | 6.89 | **0.028** |
| Management:Time:pH.std | 8 | 0.10 | 0.754 | 9 | 3.47 | 0.095 |
| Management:Time:SOM.std | 8 | 1.66 | 0.234 | [-] | [-] | [-] |
| Management:pH.std:SOM.std | [-] | [-] | [-] | 9 | 3.28 | 0.103 |
| Time:pH.std:SOM.std | 8 | 2.86 | 0.129 | [-] | [-] | [-] |
| Management:Time:pH.std:SOM.std | [-] | [-] | [-] | [-] | [-] | [-] |
| *PCOA2 Clay* |  |  |  |  |  |  |
| Management | 34 | 0.64 | 0.428 | 28 | 21.80 | **<0.0001** |
| Time | 34 | 0.15 | 0.697 | 28 | 5.67 | **0.024** |
| pH.std | 34 | 3.79 | 0.060 | 28 | 2.15 | 0.154 |
| SOM.std | 34 | 4.85 | **0.035** | 28 | 4.49 | **0.043** |
| Management:Time | 34 | 1.60 | 0.214 | 28 | 1.98 | 0.171 |
| Management:pH.std | 34 | 0.09 | 0.764 | 28 | 0.00 | 0.969 |
| Management:SOM.std | [-] | [-] | [-] | 28 | 0.40 | 0.530 |
| Time:pH.std | 34 | 0.05 | 0.831 | 28 | 0.89 | 0.353 |
| Time:SOM.std | [-] | [-] | [-] | 28 | 0.31 | 0.585 |
| pH.std:SOM.std | 34 | 1.67 | 0.205 | 28 | 2.02 | 0.166 |
| Management:Time:pH.std | 34 | 1.86 | 0.181 | 28 | 0.39 | 0.536 |
| Management:Time:SOM.std | [-] | [-] | [-] | 28 | 0.35 | 0.557 |
| Management:pH.std:SOM.std | [-] | [-] | [-] | 28 | 1.77 | 0.194 |
| Time:pH.std:SOM.std | [-] | [-] | [-] | 28 | 0.57 | 0.457 |
| Management:Time:pH.std:SOM.std | [-] | [-] | [-] | 28 | 3.37 | 0.077 |
| *PCOA2 Sand* |  |  |  |  |  |  |
| Management | 7 | 6.75 | **0.036** | 7 | 14.52 | **0.007** |
| Time | 7 | 0.62 | 0.459 | 7 | 8.48 | **0.023** |
| pH.std | 7 | 6.37 | **0.040** | 7 | 7.87 | **0.026** |
| SOM.std | 7 | 8.80 | **0.021** | 7 | 4.71 | 0.067 |
| Management:Time | 7 | 0.26 | 0.629 | 7 | 0.72 | 0.423 |
| Management:pH.std | 7 | 0.62 | 0.457 | 7 | 2.47 | 0.160 |
| Management:SOM.std | 7 | 0.77 | 0.410 | 7 | 1.75 | 0.227 |
| Time:pH.std | 7 | 5.45 | 0.052 | 7 | 0.03 | 0.869 |
| Time:SOM.std | 7 | 1.58 | 0.249 | 7 | 6.37 | **0.040** |
| pH.std:SOM.std | 7 | 23.45 | **0.002** | 7 | 4.63 | 0.068 |
| Management:Time:pH.std | 7 | 4.00 | 0.086 | 7 | 0.42 | 0.536 |
| Management:Time:SOM.std | 7 | 0.31 | 0.595 | 7 | 0.08 | 0.786 |
| Management:pH.std:SOM.std | 7 | 5.73 | **0.048** | 7 | 4.77 | 0.065 |
| Time:pH.std:SOM.std | 7 | 1.48 | 0.264 | 7 | 3.63 | 0.099 |
| Management:Time:pH.std:SOM.std | [-] | [-] | [-] | [-] | [-] | [-] |

**
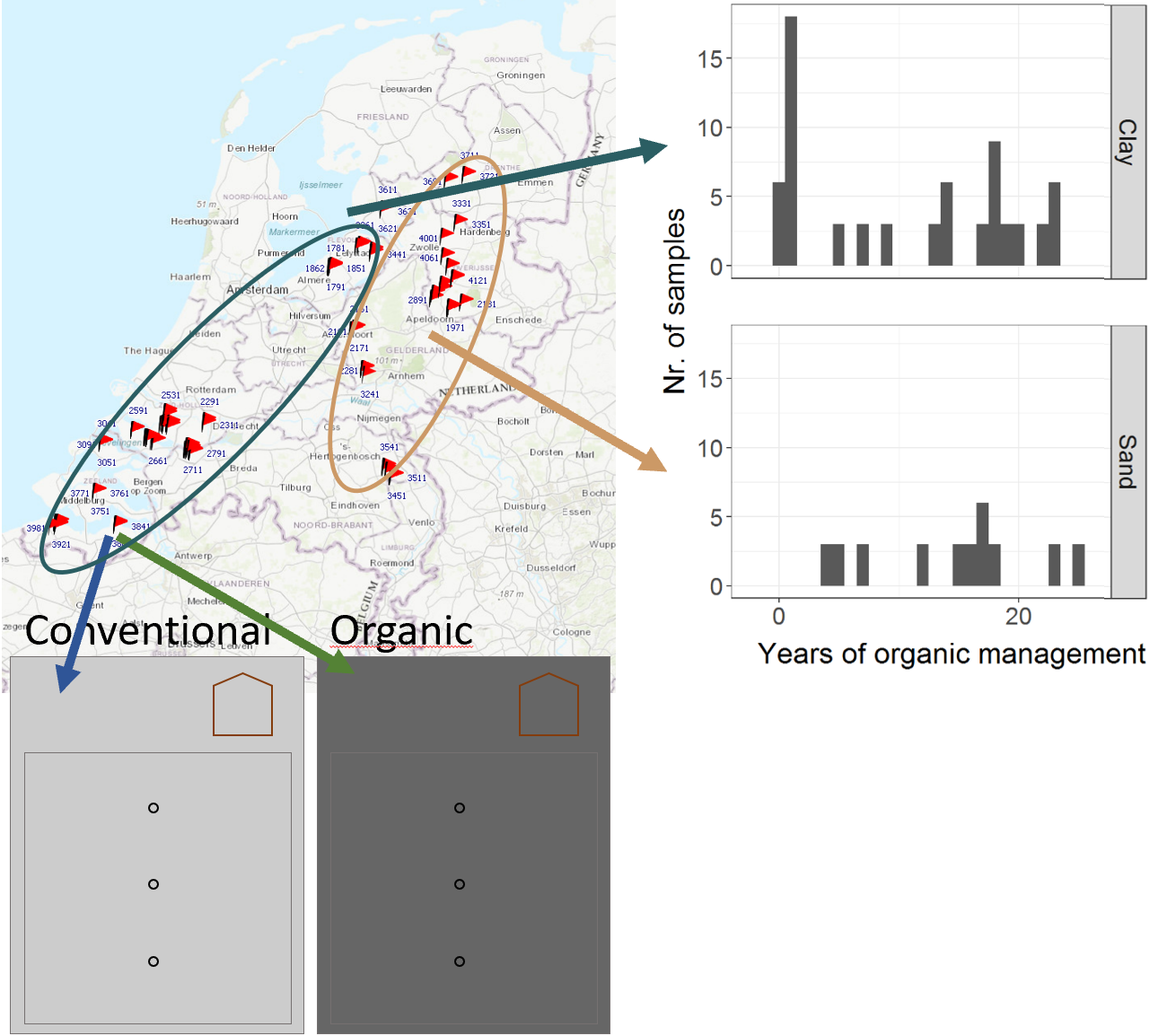
**

**Figure S1**. Sampling design of the study. Each flag is a sampling location. The blue ellipse contains the sample locations on clay soil, the yellow ellipse contians those on sandy soil. At the right there is the division of samples over time (how long the organically managed fields are under organic management). Each organic field was paired with a nearby conventional field for local reference. In each field three soil samples are taken, minimally 15 meters apart.


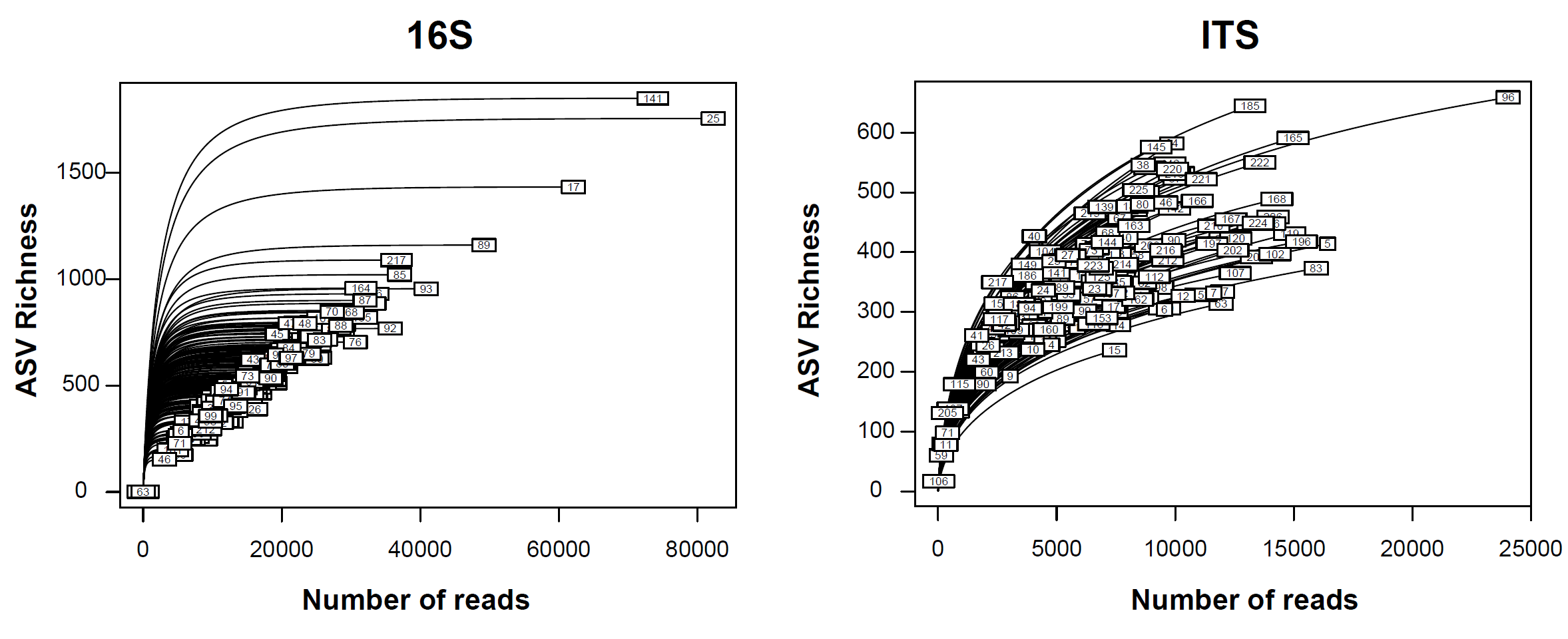
 **Fig S2.** Sample-based rarefaction curves for 16S and ITS data showing accumulated sampled diversity.


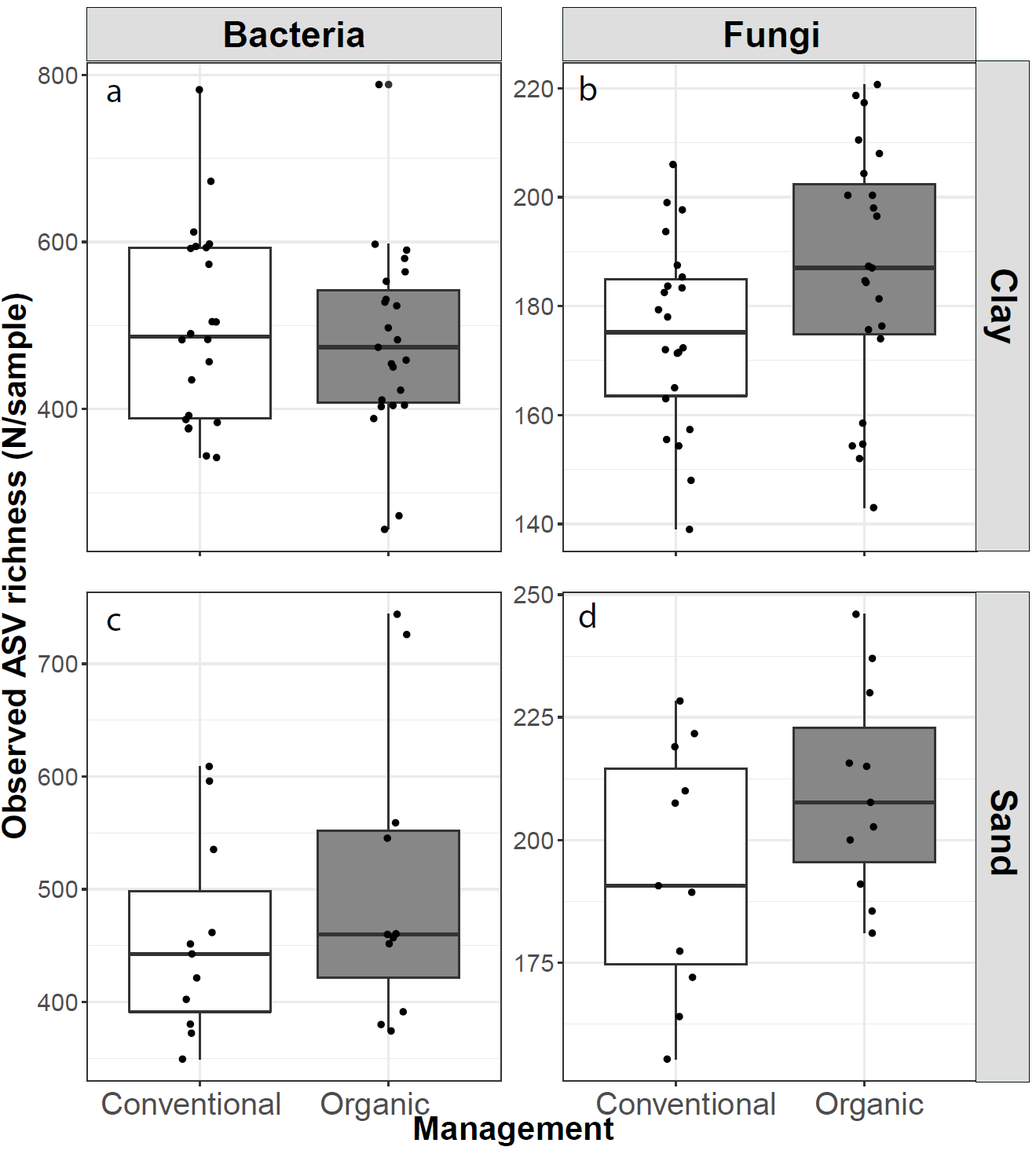


**Fig S3.** Boxplots of bacterial and fungal ASV richness in clayey and sandy soils under conventional and organic management. The median is represented by the thick horizontal line; the box is defined by the 25th and 75th percentiles (lower and upper quartile). The line is 1.5 times the spread of the box. Corresponding statistics from a linear mixed effects model can be found in Table 1.


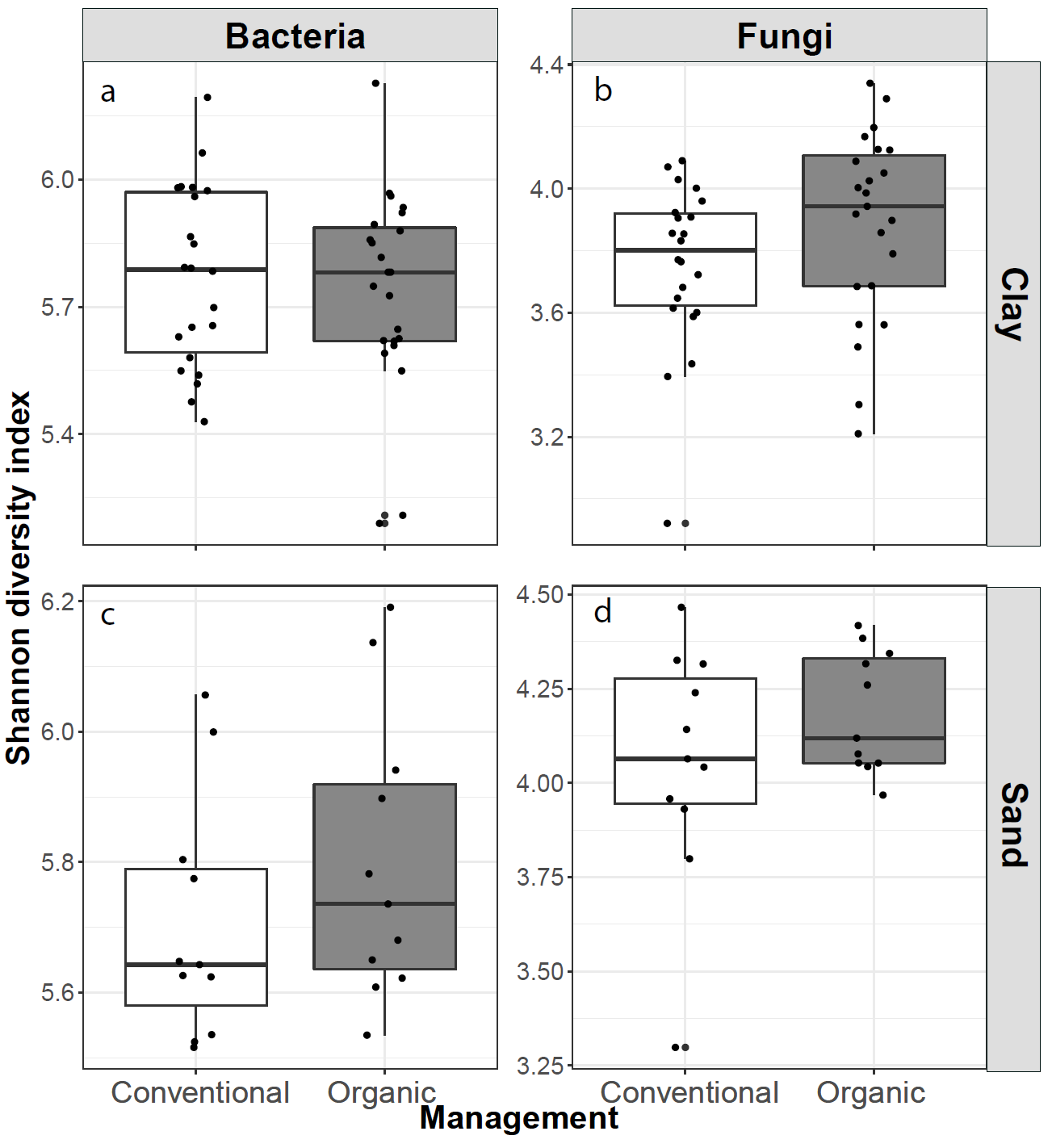
**Fig S4.** Boxplots of bacterial and fungal Shannon diversity index of clayey and sandy soils under conventional and organic management. The median is represented by the thick horizontal line; the box is defined by the 25th and 75th percentiles (lower and upper quartile). The line is 1.5 times the spread of the box. Corresponding statistics from a linear mixed effects model can be found in Table 1.


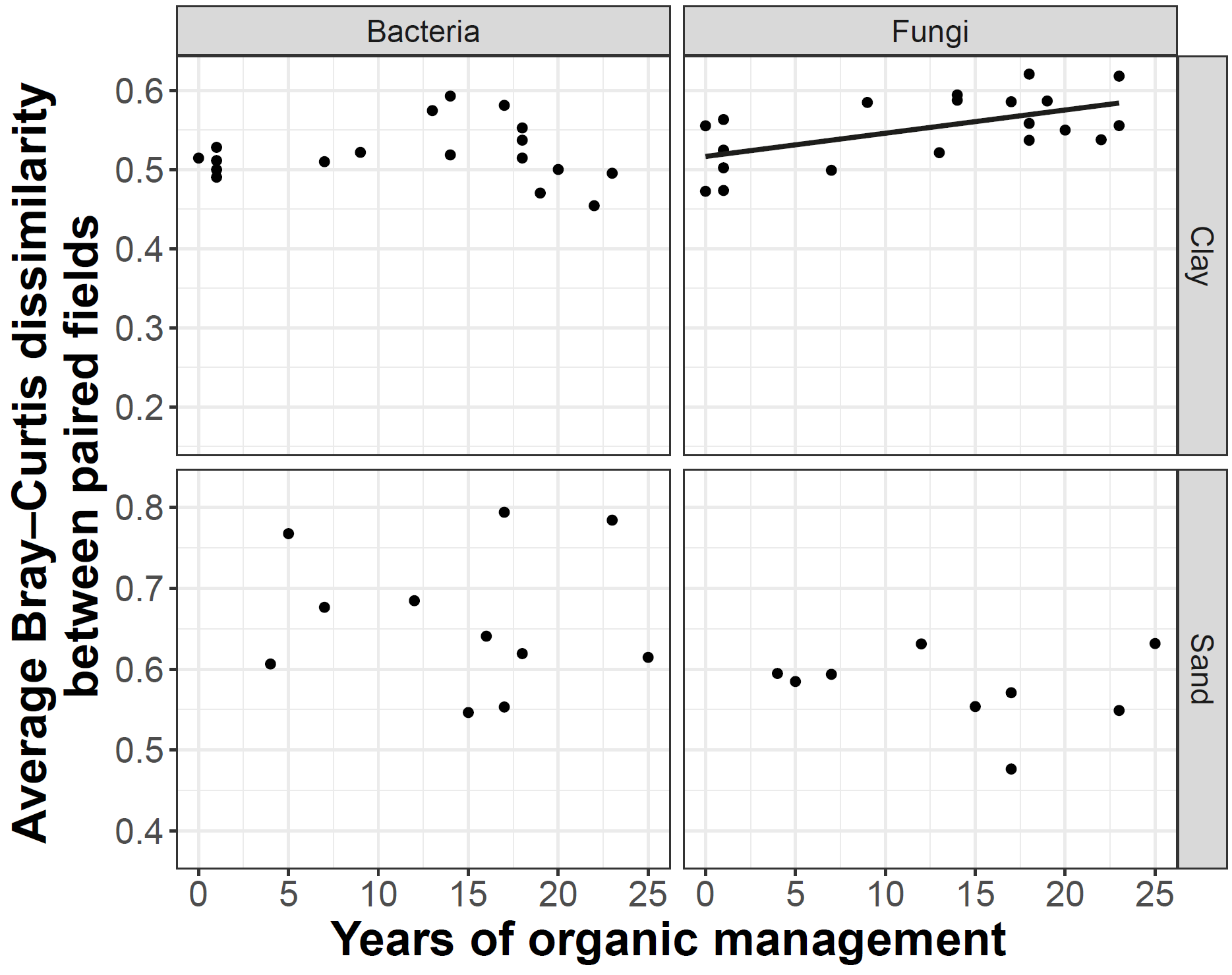


**Fig S5.** Average Bray-Curtis distance between paired organic and conventional fields related to time since conversion of the organic field. Relations were tested by linear mixed effect models with region as random factor (for clayey sites) and linear models (for sandy sites). For fungi on clayey soils there was a significant relation between Bray-Curtis dissimilarity and time since conversion (LMER: F_1,18_ = 10.0, P = 0.0053). The other relations were not significant (P > 0.20).


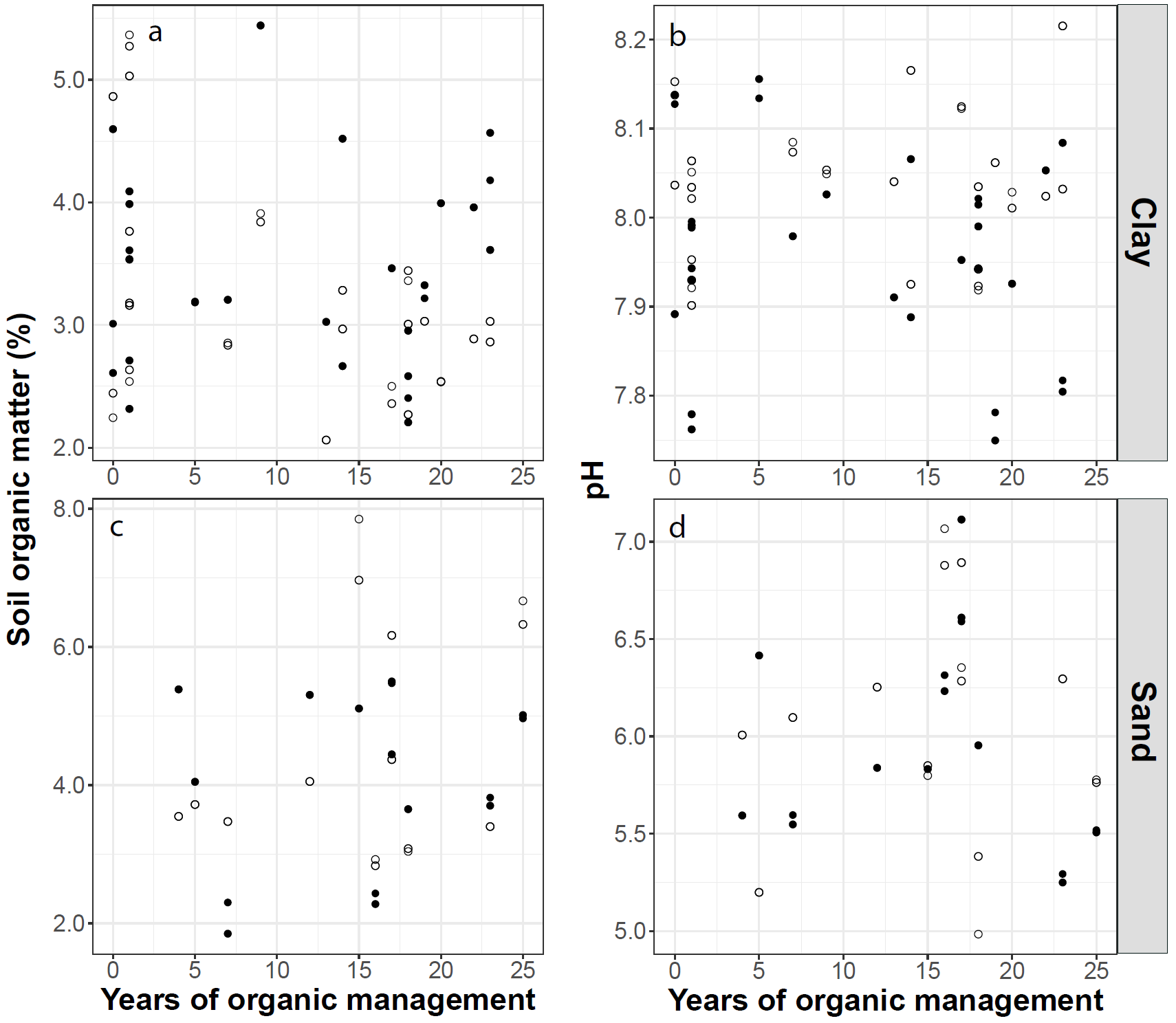


**Fig S6.** Effect of time since conversion from conventional to organic management on soil organic matter content and pH for clayey and sandy soils. Conventional fields were plotted using the time since conversion from their paired organic field. Note that the scale of the y-axes differ between soils. Relationships are tested using linear mixed models, with region as a random factor for clayey soils. Numerator degrees of freedom is 1 for all variables; denominator degrees of freedom is 40 for clayey and 18 for sandy soils (marine clay: SOM: F = 1.19, P = 0.28; pH: F = 0.13, P = 0.71; sand: SOM: F = 0.95, P = 0.34; pH: F = 0.09, P = 0.77).


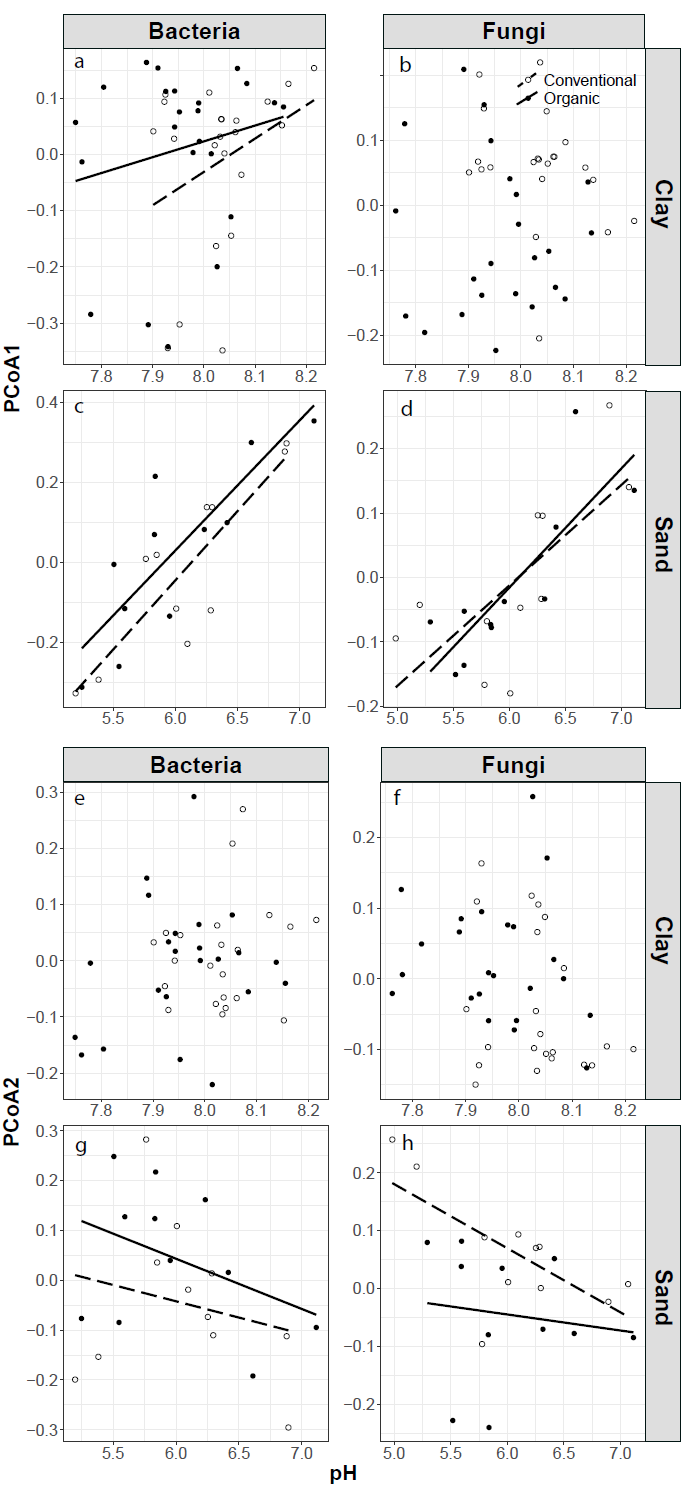


**Fig S7.** Effect of pH on bacterial, archaeal and fungal composition as represented by PCoA1 and PCoA2. Relationship or correlation of soil pH with bacterial and fungal community composition as resolved by PCoA axes were tested using linear mixed models, with region as a random factor for clayey soils. Lines are plotted for significant relationships only. Solid lines correspond to organic management, dashed lines correspond to conventional management. Corresponding statistics can be found in Table S2.


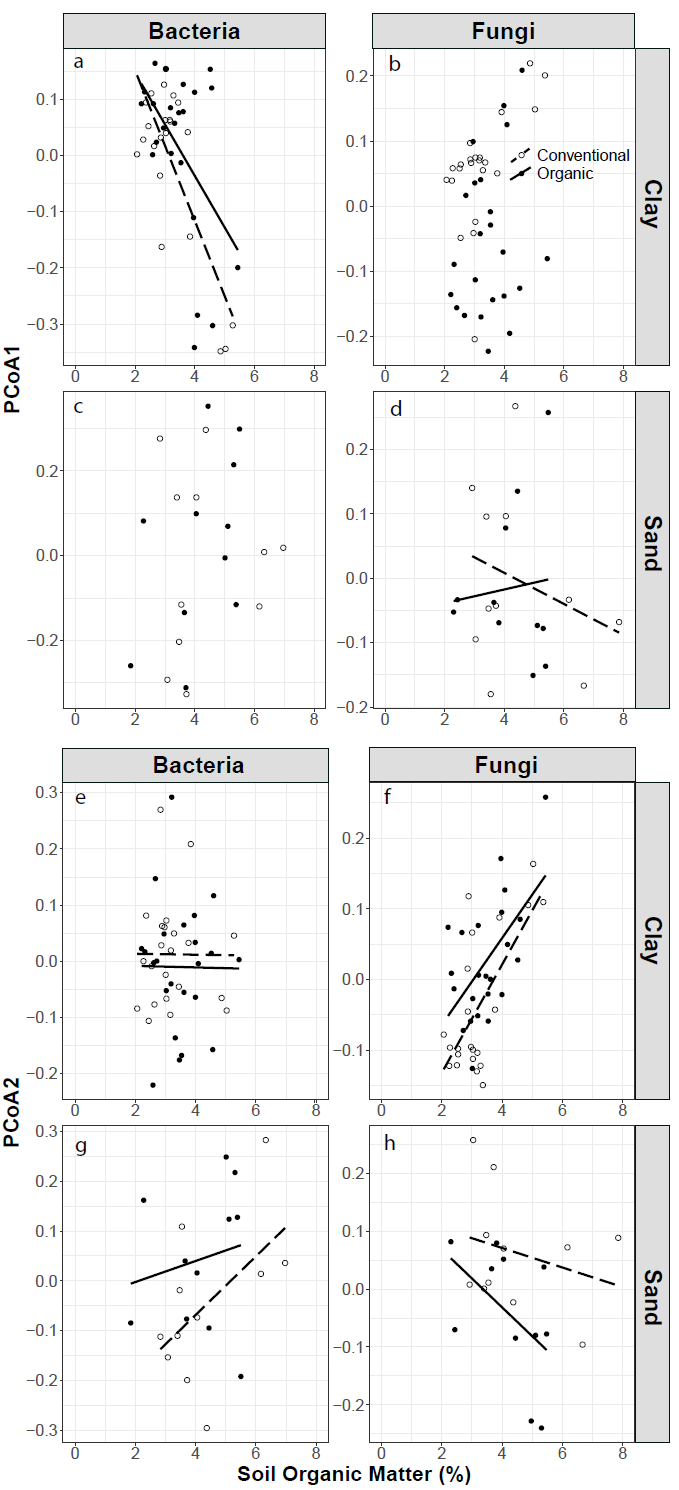


**Fig. S8.** Effect of Soil Organic Matter (SOM) content on bacterial, archaeal and fungal composition as represented by PCoA1 and PCoA2. Relationships are tested using linear mixed models, with region as a random factor for marine clay soils. Lines are plotted for significant relationships only. Solid lines correspond to organic management, dashed lines correspond to conventional management. Corresponding statistics can be found in Table S2.
